# 1039. Rapid Restoration of Bile Acid Compositions After Treatment with RBX2660 for Recurrent *Clostridioides difficile* Infection—Results from the PUNCH CD3 Phase 3 Trial

**DOI:** 10.1093/ofid/ofab466.1233

**Published:** 2021-12-04

**Authors:** Romeo Papazyan, Bryan Fuchs, Ken Blount, Carlos Gonzalez, Bill Shannon

**Affiliations:** 1 Ferring Research Institute, San Diego, CA; 2 Rebiotix, Inc., Roseville, Minnesota; 3 BioRankings, LLC, St. Louis, Missouri

## Abstract

**Background:**

Microbiota-based treatments are increasingly evaluated as a strategy to reduce recurrence of *Clostridioides difficile* infection (rCDI), and their proposed mechanisms include restoration of the microbiota and microbiota-mediated functions, including bile acid metabolism. RBX2660—a broad-consortium investigational live biotherapeutic—has been evaluated in >600 participants in 6 clinical trials, with consistent reduction of rCDI recurrence. Here we report that fecal bile acid compositions were significantly restored in treatment-responsive participants in PUNCH CD3—a Phase 3 randomized, double-blinded, placebo-controlled trial of RBX2660.

**Methods:**

PUNCH CD3 participants received a single dose of RBX2660 or placebo between 24 to 72 hours after completing rCDI antibiotic treatment. Clinical response was the absence of CDI recurrence at eight weeks after treatment. Participants voluntarily submitted stool samples prior to blinded study treatment (baseline), 1, 4 and 8 weeks, 3 and 6 months after receiving study treatment. A liquid chromatography tandem mass spectrometry method was developed to extract and quantify 33 bile acids from all participant fecal samples received up to the 8-week time point. Mean bile acid compositions were fit to a Dirichlet multinomial distribution and compared across time points and between RBX2660- and placebo-treated participants.

**Results:**

Clinically, RBX2660 demonstrated superior efficacy versus placebo (70.4% versus 58.1%). RBX2660-treated clinical responders’ bile acid compositions shifted significantly from before to after treatment. Specifically, primary bile acids predominated before treatment, whereas secondary bile acids predominated after treatment (Figure 1A). These changes trended higher among RBX2660 responders compared to placebo responders. Importantly, median levels of lithocholic acid (LCA) and deoxycholic acid (DCA) showed large, significant increases after treatment (Figure 1B).

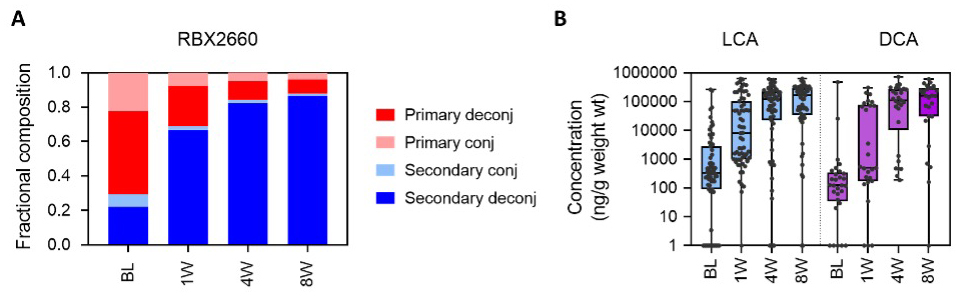

A. Bile acid compositions before (BL) and up to 8 weeks after RBX2660 treatment among treatment responders. Compositions are shown as the fraction of total bile acids classified as primary or secondary conjugated or deconjugated bile acids. B. Concentrations of lithocholic acid (LCA) and deoxycholic acid (DCA) among RBX2660 treatment responders, shown with individual samples and time point group median with interquartile ranges.

**Conclusion:**

Among PUNCH CD3 clinical responders, RBX2660 significantly restored bile acids from less to more healthy compositions. These clinically correlated bile acid shifts are highly consistent with results from a prior trial of RBX2660.

**Disclosures:**

**Romeo Papazyan, PhD**, **Ferring Research Institute** (Employee) **Bryan Fuchs, PhD**, **Ferring Pharmaceuticals** (Employee) **Ken Blount, PhD**, **Rebiotix Inc., a Ferring Company** (Employee)

